# Operative and Hepatic Function Outcomes of Laparoscopic vs. Open Liver Resection: A Systematic Review and Meta-Analysis

**DOI:** 10.7759/cureus.47274

**Published:** 2023-10-18

**Authors:** Reda H Mithany, Farid Gerges, M Hasaan Shahid, Shenouda Abdallah, Mina Manasseh, Mark Abdelmaseeh, Mazin Abdalla, Eiad Elmahi

**Affiliations:** 1 Laparoscopic Colorectal Surgery, Kingston Hospital National Health Services (NHS) Foundation Trust, Kingston, GBR; 2 General and Emergency Surgery, Kingston Hospital National Health Services (NHS) Foundation Trust, Kingston, GBR; 3 Surgery, Postgraduate Medical Institute, Lahore, PAK; 4 Surgery, Jaber Al-Ahmad Hospital, Kuwait, KWT; 5 General Surgery, Torbay and South Devon National Health Services (NHS) Foundation Trust, Torquay, GBR; 6 General Surgery, Faculty of Medicine, Assuit University, Assuit, EGY; 7 General Surgery, Kingston Hospital National Health Services (NHS) Foundation Trust, Kingston, GBR; 8 General Surgery, Lincoln County Hospital, Lincoln, GBR

**Keywords:** systematic review, hepatectomy complications, “post-hepatectomy bleeding”, open liver resection, laparoscopic liver resection

## Abstract

Liver resection is a pivotal treatment for various liver diseases, and the choice between laparoscopic (LR) and open (OR) methods is debatable. This study aims to compare their respective complications and hepatic outcomes comprehensively, providing critical insights to guide clinical decisions and optimize patient results.

We conducted a comprehensive review across PubMed, SCOPUS, WOS, and the Cochrane Library until September 2023. Randomized controlled trials (RCTs) comparing laparoscopic (LR) and open (OR) liver resections were included. Data screening, extraction, and quality assessments utilized the Risk of Bias (ROB-2). We conducted our analysis using Review Manager (RevMan 5.4) software, and the data were presented as risk ratios (RR) and mean differences (MD) with 95% confidence intervals (CI).

Our comprehensive research yielded 3,192 relevant records, and 9 RCTs were finally included. LR exhibited reduced operative bleeding (MD = -82.87 ml, 95% CI: -132.45 to -33.30, P=0.001) and shorter hospital stays (MD = -2.32 days, 95% CI: -3.65 to -0.98, P=0.0007). The risk of complications was significantly lower in the LR group (RR = 0.57, 95% CI: 0.43-0.76, P<0.0001), especially in Clavian-Dindo classification degree 1 and 2 complications (RR = 0.47, 95% CI: 0.28-0.79, P=0.005). LR patients also had lower postoperative AST levels at one day (MD = -123.16 U/L, 95% CI: -206.08 to -40.24, P=0.004) and three days (MD = -35.95 U/L, 95% CI: -65.83 to -6.06, P=0.02).

These findings underscore LR's superiority, emphasizing its potential to significantly enhance patient outcomes, reduce complications, and improve recovery in liver resection procedures.

## Introduction and background

Liver resection represents a surgical procedure aimed at removing a specific segment or portion of the liver, and it has become a cornerstone in treatments for various liver diseases [1]. This intervention addresses hepatic pathologies by excising the affected tissue while sparing healthy liver parenchyma, optimizing postoperative liver function [2].

The utility of liver resection extends across a broad spectrum of clinical scenarios. Beyond its well-established role in managing malignant liver tumors, including hepatocellular carcinoma (HCC), cholangiocarcinoma, and colorectal liver metastases, liver resection is equally valuable in addressing benign conditions, such as liver cysts and certain metabolic liver disorders [3,4]. Additionally, sometimes liver resection is essential in managing trauma-related liver injuries and is particularly significant in pediatric hepatoblastoma cases [5,6].

Laparoscopic liver resection (LR) represents a minimally invasive approach that employs smaller incisions and the utilization of a laparoscope for visualization [7]. This approach offers several advantages, including reduced postoperative pain, shorter hospital stays, faster recovery times, and improved cosmetic outcomes. Nevertheless, its application may be limited to smaller lesions and necessitate advanced surgical skills and specialized equipment [8,9].

Traditional open liver resection (OR), the conventional surgical approach, necessitates a larger abdominal incision, providing surgeons with improved visibility and control, especially for dealing with sizable tumors and intricate cases. Despite its advantages, this method is linked to extended recovery periods, heightened postoperative discomfort, and a greater likelihood of complications [10].

In recent years, the medical literature has witnessed an increasing number of studies comparing LR and OR techniques. These studies have sought to comprehensively evaluate various aspects, including operative outcomes, postoperative complications, length of hospital stay, and long-term survival. While some reports suggest that laparoscopic liver resection may benefit patient recovery and reduce postoperative complications, others contend that OR remains the gold standard for more complex and advanced cases [11,12].

We aim to comprehensively evaluate operative complications and hepatic function outcomes in LR versus OR patients. Our findings offer a more robust and evidence-based comparison of these two surgical approaches. Ultimately, our work aspires to contribute valuable insights that can inform clinical decision-making and enhance patient outcomes within liver resection procedures.

## Review

Material and method

The Cochrane Handbook for Systematic Reviews and Interventions [13] and Preferred Reporting Items for Systematic Reviews and Meta-Analyses (PRISMA) standards [14] were adhered to throughout this meta-analysis.

Search Strategy

Our search encompassed various electronic databases, including PubMed, SCOPUS, Web of Science (WOS), and the Cochrane Library, from the inception of each database to September 2023. The search strategy involved a combination of keywords such as "laparoscopic liver resection," "open liver resection," "hepatectomy," "liver surgery," "operative complications," and "hepatic function." We included randomized controlled trials (RCTs) that directly compare LR and OR. Additionally, we focused on studies published in English and excluded case reports, reviews, abstracts, conference proceedings, cohort studies, and those with small sample sizes (<10 patients per group).

Data Screening and Extraction

Two independent reviewers screened the titles and abstracts of identified articles to determine eligibility, and the full texts of potentially relevant papers were assessed for inclusion. Discrepancies were resolved through consensus or consultation with a third reviewer. Data extraction followed a standardized form, collecting information on study characteristics and patient demographics. Quality assessment of included studies was performed using appropriate tools, such as the Newcastle-Ottawa Scale for cohort studies.

Quality Assessment

The Risk of Bias (ROB) tool, version 2, was used to assess the studies' bias in this meta-analysis [15]. The tool evaluates five domains: bias caused by the randomization technique, bias caused by variations from planned interventions, bias caused by missing outcome data, bias in outcome assessment, and bias in the selection of the reported result. For each domain, the risk of bias was rated as low, moderate, or high. Using ROB 2, two reviewers independently evaluated each research’s bias risk. Any differences were worked out via debate and consensus.

Statistical Analysis

Statistical analysis was conducted using the Review Manager (RevMan 5.4) software. We calculated risk ratios (RRs) with 95% confidence intervals (CIs) for dichotomous outcomes. We computed mean differences (MDs) with 95% CIs for continuous outcomes. To assess heterogeneity among studies, we employed the p statistic and used a random-effects model for substantial heterogeneity (p < 0.1), while a fixed-effects model was applied otherwise.

Results

**Figure 1:**
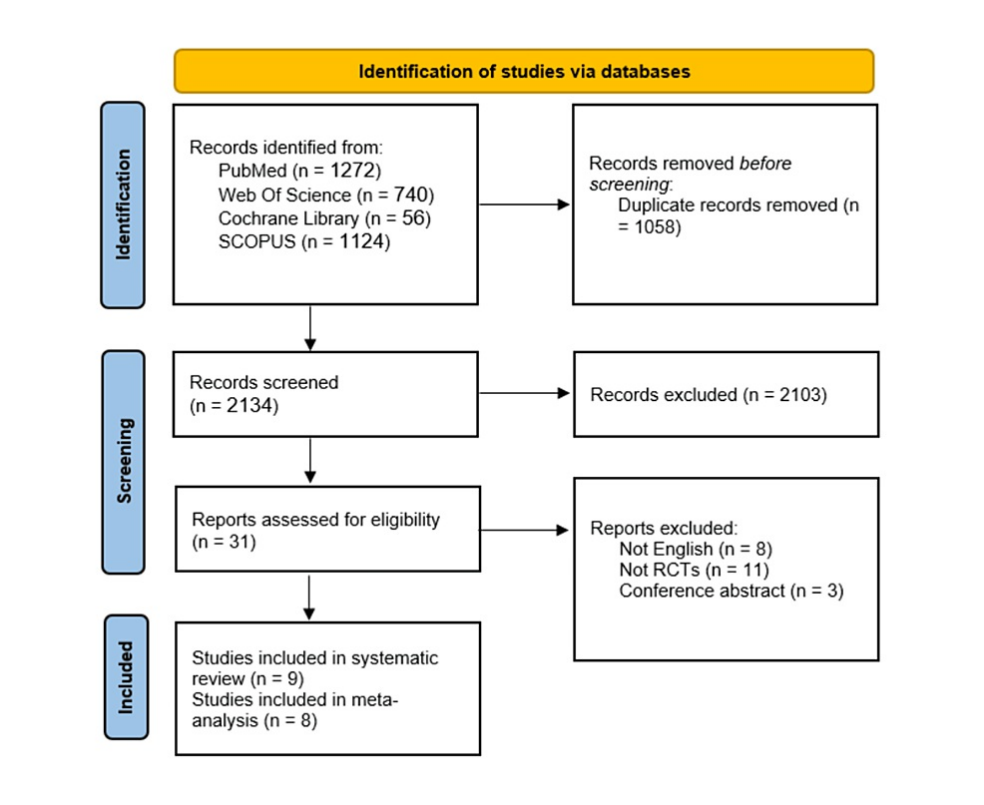
PRISMA flow diagram

Our comprehensive research yielded 3,192 relevant records. After removing 1058 duplicate records, 2134 underwent screening and initial screenings. After the title and abstract screening, 2013 studies were excluded. Finally, 31 reports were thoroughly evaluated for eligibility. Among these, nine studies were included in the systematic review [16-24], and eight were included in the meta-analysis [17-24] (Figure 1).

Baseline Characteristics

The studies encompassed a global scope, spanning countries like Egypt, Spain, and Norway, and a significant portion was conducted in China. Interestingly, the age range of the patients was quite consistent, falling between 47.8 and 67 years. Moreover, male participants comprised the majority of the study cohorts. The studies included liver cancer patients, colorectal liver metastases, and hepatolithiasis. Further details are shown in Tables 1, 2.

**Table 1:** Summary of the included studies LR: Laparoscopic liver resection, OR: Open liver resection

Study ID	Site	Study protocol	Patients	Primary outcomes	Conclusion
Ding et al. 2015	China	Local committee	Hepatolithiasis within the left lateral lobes	Operative and complications outcomes	They reported that LR for left lateral hepatolithiasis was as safe as open surgery but offered operating time, bleeding control, and hospital stay advantages.
El-Gendi et al. 2017	Egypt	Local committee	Solitary Hepatocellular Carcinoma Less Than 5 cm in Cirrhotic Patients	Operative and complications outcomes	LHR outperformed OR, with significantly shorter hospital stays and no compromise in oncological outcomes.
Fretland et al. 2017	Norway	NCT01516710	Colorectal liver metastases	Complications	For patients undergoing parenchyma-sparing liver resection for colorectal metastases, laparoscopic surgery resulted in significantly fewer postoperative complications than open surgery.
Jiang et al. 2015	China	NR	Primary Hepatic Carcinoma	Hepatic function	LR could enhance perioperative outcomes for patients with primary hepatic carcinoma compared to OR.
Li et al. 2015	China	Local committee	Hepatocellular carcinoma	Inflammatory markers	The findings demonstrated that laparoscopic surgery patients had reduced secretion levels of IL-6 and IL-8.
Robles-Campos et al. 2019	Spain	NCT02727179	Colorectal liver metastases	Complications	LR showed comparable oncological outcomes and offered the benefits of favorable short-term results.
Sun et al. 2017	China	Local committee	Liver cancer patient	Operative and Hepatic function outcomes	Laparoscopic hepatectomy for liver cancer demonstrated clear clinical effectiveness, minimal trauma, high safety, low complication rates, minimal impact on patients' cellular immune function, and swift postoperative recovery.
Wong-Lun-Hing et al. 2017	Multinational	NCT00874224	Colorectal liver metastases and others	Operative outcomes	The study was not able to conclude on time to functionally recover.
Yan et al. 2020	China	Local committee	Hepatocellular Carcinoma	Value of Serum LHPP-associated miR-765	Patients with a low miR-765 level had the option of both LR and OR, but LH was the preferred recommendation for others.

**Table 2:** Baseline characteristics of the included studies. BMI: Body mass index, ASA: American Society of Anesthesiologists, LR: Laparoscopic liver resection, OR: Open liver resection, M: Mean, SD: Standard deviation

Study ID	Study arms	Sample	Age, M ± SD	Gender, Male (%)	BMI, Kg/m2, M ± SD	ASA, II/II/III/IV	Tumor size (cm), M ± SD
Ding et al. 2015	LR	49	57.53 ± 6.31	26 (53%)	-	-	-
	OR	49	58.42 ± 7.21	27 (55%)	-	-	-
El-Gendi et al. 2017	LR	25	54.52 ± 7.01	16 (64%)	28.96 ± 1.83	0/20/5/0	3.33 ± 0.57
	OR	25	54.20 ± 7.41	14 (56%)	27.96 ± 2.03	0/21/4/0	3.38 ± 0.59
Fretland et al. 2017	LR	133	67 ± 8	77 (65%)	26 ± 5	11/59/51/1	-
	OR	147	66 ± 10	87 (54%)	25 ± 4	20/73/44/0	-
Jiang et al. 2015	LR	50	55.4 ± 2.62	35 (70%)	22.31 ± 2.85	-	3.18 ± 0.29
	OR	50	56.55 ± 1.87	37 (74%)	24.27 ± 3.47	-	3.22 ± 0.31
Li et al. 2015	LR	12	49.25 ± 12.99	12 (100%)	-	-	5.96 ± 2.82
	OR	14	55 ± 11.6	14 (100%)	-	-	6.23 ± 3.16
Robles-Campos et al. 2019	LR	96	65.33 ± 10.54	61 (63.5%)	27 ± 3.01	1/43/52/0	3.33 ± 0.75
	OR	97	67 ± 15.05	71 (73.2%)	27.7 ± 4.52	1/50/46/0	3.7 ± 2.26
Sun et al. 2017	LR	100	48.4 ± 16.5	56 (56%)	-	-	5.35 ± 3.22
	OR	100	47.8 ± 15.4	57 (57%)	-	-	5.42 ± 3.18
Wong-Lun-Hing et al. 2017	LR	13	65 ± 15	9 (69%)	27 ± 2.66	1/9/3/0	-
	OR	11	60 ± 15.3	5 (38.5%)	29.4 ± 7.13	3/7/1/0	-
Yan et al. 2020	LR	80	-	107 (67%)	-	-	-
	OR	80	-		-	-	-

Quality Assessment

**Figure 2:**
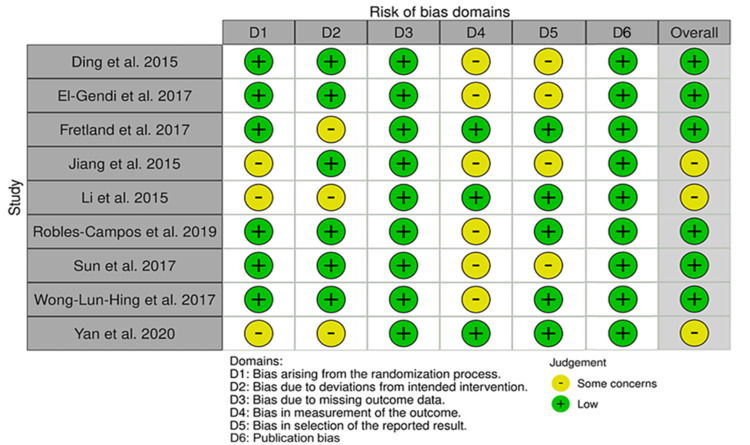
Risk of bias (ROB-2) graph

**Figure 3:**
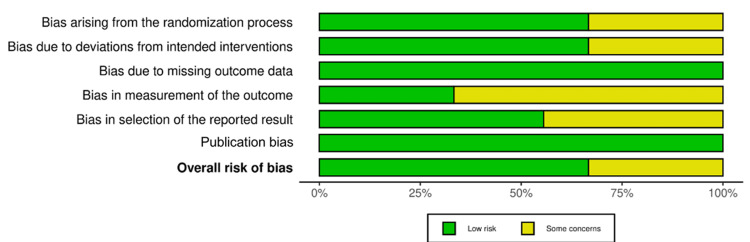
Risk of bias (ROB-2) summary

The quality of the included studies varied, and three studies [16,20,21] were rated as having “some concerns.” Li et al. (2015) and Yan et al. (2020) showed “some concerns” in the randomization process and deviation from intended intervention domains. Jiang et al. (2015) had “some concerns” in the randomization process, outcome measurement, and reported result selection domains. The rest of the studies were judged as having an overall risk of bias (Figures 2, 3).

Operating Time

**Figure 4:**
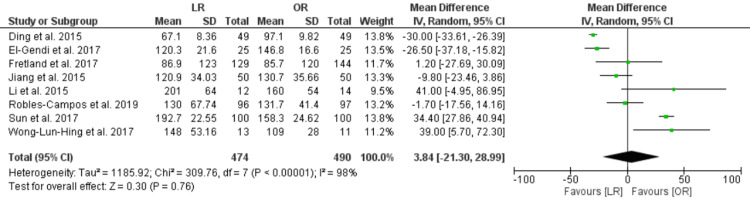
Operative time, minutes

The pooled analysis of eight RCTs showed no significant difference between LR and OR in the operating time [MD = 3.84, CI 95% (-21.3, 28.99), P = 0.76]. The data were heterogenous (P < 0.00001, I² = 98%), and this heterogeneity could not be resolved (Figure 4).

Amount of Bleeding (ml)

**Figure 5:**
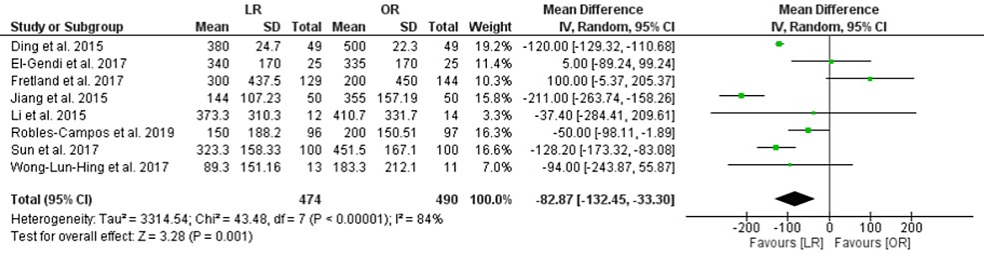
Intraoperative blood loss, ml

The amount of operative bleeding was less in the LR group compared with the OR group [MD = -82.87, CI 95% (-132.45, -33.30), P = 0.001]. The data were heterogenous (P < 0.00001, I² = 84%), and this heterogeneity could not be resolved (Figure 5).

Length of Hospital Stay (Days)

**Figure 6:**
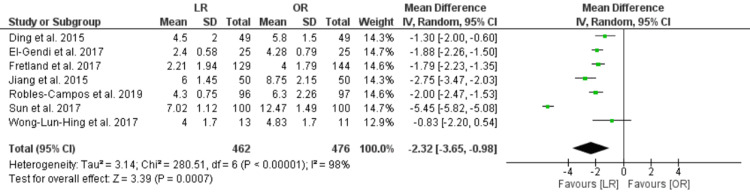
Length of hospital stay, days

The length of hospital stay in the LR group was shorter than in the OR group [MD = -2.32, CI 95% (-3.65, -0.98), P = 0.0007]. The data showed unresolved heterogeneity (P < 0.00001, I² = 98%) (Figure 6).

Intensive Care Unit (ICU) Need

There was no significant difference between LR and OR in the need for ICU admission [RR = 1.07, CI 95% (1.07, 4.08), P = 0.92], and the data were homogenous (p = 0.14, I² = 53%) (Table 3).

**Table 3:** Secondary outcomes summary ICU: Intensive care unit, CI: Confidence interval, Bold numbers refer to significant p-values

Outcome	No. of studies	Sample	Statistical Method	Effect Estimate	P-value
1. ICU need	2	319	Risk Ratio (M-H, Fixed, 95% CI)	1.07 [0.28, 4.08]	0.92
2. Additional procedure during operation					
2.1. Blood transfusion	3	267	Risk Ratio (M-H, Fixed, 95% CI)	0.41 [0.15, 1.13]	0.08
2.2. Associated cholecystectomy	3	267	Risk Ratio (M-H, Fixed, 95% CI)	0.59 [0.37, 0.94]	0.03
3. Clavian-Dindo classification					
3.1. Degrees 1 and 2	4	540	Risk Ratio (M-H, Fixed, 95% CI)	0.47 [0.28, 0.79]	0.005
3.2. Degrees 3 and 4	4	540	Risk Ratio (M-H, Fixed, 95% CI)	0.60 [0.34, 1.04]	0.07
4. Bile leak	2	466	Risk Ratio (M-H, Fixed, 95% CI)	1.76 [0.24, 13.06]	0.58
5. Pleural effusion	2	466	Risk Ratio (M-H, Fixed, 95% CI)	0.68 [0.13, 3.48]	0.64
6. Ascites	2	243	Risk Ratio (M-H, Fixed, 95% CI)	0.60 [0.15, 2.37]	0.47
7. Pneumothorax	2	466	Risk Ratio (M-H, Fixed, 95% CI)	1.40 [0.35, 5.63]	0.63
8. Urinary tract infection	2	466	Risk Ratio (M-H, Fixed, 95% CI)	1.09 [0.25, 4.77]	0.91
9. Post-operative AST (U/L)					
9.1. After one day	2	300	Mean Difference (IV, Random, 95% CI)	-123.16 [-206.08, -40.24]	0.004
9.2. After 3 days	2	300	Mean Difference (IV, Random, 95% CI)	-35.95 [-65.83, -6.06]	0.02
10. Post-operative ALT (U/L)					
10.1. After one day	2	300	Mean Difference (IV, Random, 95% CI)	-167.68 [-232.25, -103.10]	< 0.00001
10.2. After 3 days	2	300	Mean Difference (IV, Random, 95% CI)	-87.37 [-189.35, 14.62]	0.09
11. Post-operative TBIL (μmol/L)					
10.2. After one day	2	300	Mean Difference (IV, Fixed, 95% CI)	-3.60 [-4.02, -3.18]	< 0.00001
10.2. After 4 days	2	300	Mean Difference (IV, Random, 95% CI)	-2.98 [-6.28, 0.32]	0.08
12. Post-operative ALT (U/L)					
10.3. After one day	2	300	Mean Difference (IV, Random, 95% CI)	2.30 [-2.38, 6.97]	0.34
10.2. After 5 days	2	300	Mean Difference (IV, Random, 95% CI)	3.64 [2.33, 4.95]	< 0.00001

Additional Procedure During Operation

Our pooled analysis showed no significant difference between the two groups in the need for blood transfusion [RR = 0.41, CI 95% (0.15, 1.13), P = 0.08], and the data were homogenous (P = 0.79, I² = 0). The rate of associated cholecystectomy in the LR group was lower than in the OR group [RR = 0.59, CI 95% (0.37, 0.94), P = 0.03], and the data were homogenous (P = 0.95, I² = 0) (Table 3).

Complications

**Figure 7:**
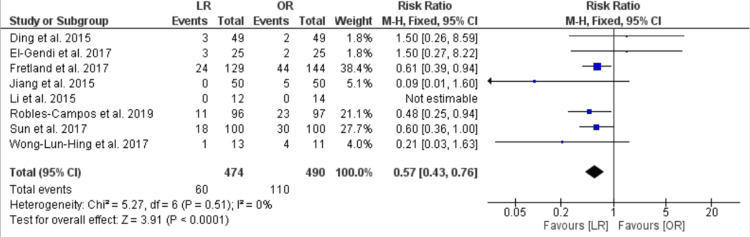
Total complications

The pooled analysis of eight RCTs showed a lower risk of complications in the LR group compared with the OR group [RR = 0.57, CI 95% (0.43, 0.76), P < 0.0001], and the data were homogenous (P = 0.51, I2 = 0) (Figure 7).

Clavian-Dindo Classification of Complications

Our pooled analysis showed a lower risk of degree 1 and 2 Clavian-Dindo complications in the LR group compared with the OR group [RR = 0.47, CI 95% (0.28, 0.79), P = 0.005], and there was no significant difference between the two groups in degree 3 and 4 [RR = 0.60, CI 95% (0.34, 1.04), P = 0.07]. The data were homogenous in both analyses (P = 0.73, I2 = 0) and (P = 0.66, I2 = 0) (Table 3).

Specific Complications

**Figure 8:**
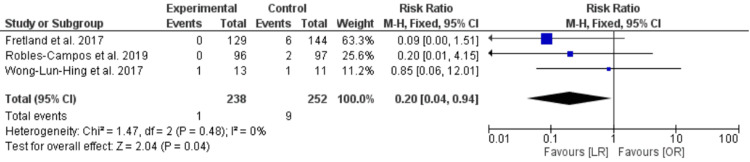
Wound infection

The risk of wound infection in the LR group was less than in the OR group [RR = 0.2, CI 95% (0.04, 0.94), P = 0.0007)], and the data was homogenous (P = 0.48, I² = 0) (Figure 8).

Other Complications

Other complications, like bile leaks, pleural effusions, ascites, pneumothorax, and urinary tract infections, did not significantly differ between the two groups (Table 3).

Liver functions

Postoperative AST (U/L)

Our pooled analysis revealed a significantly lower AST level in the LR group compared with the OR group after one and three days [MD = -123.16, CI 95% (-206.08, -40.24), P = 0.004)] and [MD = -35.95, CI 95% (-65.83, -6.06), P = 0.02)], respectively. The data showed unresolved heterogeneity in both analyses (P = 0.001, I2 = 90%) and (P = 0.002, I2 = 89%) (Table 3).

Postoperative ALT (U/L)

After one day, the post-operative ALT level in the LR group was lower than in the OR group [MD = -167.68, CI 95% (-232.25, -103.10), P < 0.00001)], but there was no significant difference between both groups after three days [MD = -87.37, CI 95% (-189.35, 14.62), P = 0.09)]. The data was heterogeneous in both analyses (P = 0.03, I2 = 78%) and (P < 0.00001, I2 = 96%), and this heterogeneity could not be resolved (Table 3).

Postoperative Albumin-Bilirubin (TBIL) (μmol/L)

The pooled analysis of two RCTs showed a significantly lower level of TBIL in the LR group compared with the OR group after one day [MD = -3.6, CI 95% (-4.02, -3.18), P < 0.00001)], and the data were homogenous (P = 0.58, I2 = 0). After three days, there was no significant difference between the two groups [MD = -2.98, CI 95% (-6.28, 0.32), P = 0.08)], but the data showed unresolved heterogeneity (P = 0.002, I2 = 90%) (Table 3).

Postoperative Albumin (g/L)

After one day, there was no significant difference between the LR and OR groups in the albumin level groups [MD = 2.3, CI 95% (-2.38, 6.97), P = 0.34)]. After three days, the albumin level in the LR groups was higher than in the OR groups [MD = 3.64, CI 95% (2.33, 4.95), P < 0.00001)]. Both analyses showed unresolved heterogeneity (P < 0.00001, I2 = 97%) (P = 0.09, I2 = 64%) (Table 3).

Discussion

In this comprehensive meta-analysis comparing LR and OR, we provided valuable insights into the surgical outcomes and postoperative parameters. Our findings underline that LR offers numerous advantages over the traditional OR method. LR demonstrated significantly reduced operative bleeding, shorter hospital stays, and a lower risk of complications. Moreover, LR exhibited superior outcomes in terms of liver function markers, notably lower postoperative AST levels. However, heterogeneity persisted in some analyses, indicating the need for further research to explore these variations. Despite this, the evidence overwhelmingly supports the efficacy and safety of laparoscopic liver resection, positioning it as a promising approach for liver surgeries.

Kabir et al.’s (2021) meta-analysis focused on patients with HCC and liver cirrhosis. They found LR to improve overall survival and perioperative outcomes significantly. Notably, their research contributed valuable data to support the efficacy of LR in cirrhotic patients with HCC [25]. Xing et al. (2020) reported similar results, and their findings aligned with ours, showing shorter hospital stays, decreased blood loss, and lower complication rates with LR. While they reported comparable overall survival, their study emphasized the benefits of LR for disease-free survival, providing essential insights into long-term outcomes [26].

Zhou et al. (2013) and Zhou et al. (2015) concentrated on colorectal liver metastasis and compared LR with OR [27,28]. Their findings reinforced our results, showcasing reduced blood loss and postoperative complications with LR. Importantly, their study provided evidence of LR’s safety and efficacy in CLM cases, corroborating our observations.

Leong et al. (205) also showed superiority in reduced blood loss, fewer transfusions, and shorter hospital stays. Importantly, they reaffirmed the safety and viability of LLR for curative resection of HCC [29].

The most recent meta-analysis by Haney et al. (2021) observed reduced complications, shorter hospital stays, and lower blood loss in LLR patients, reinforcing the procedure's advantages. However, it's crucial to note that some of the included studies' references weren’t reachable, potentially impacting the study's reliability. Also, they included abstracts that might lack detailed methodology and results, leading to incomplete conclusions [30]. 

Future enhancements in this field may involve advancements in robotic training fellowships, standardization of surgical techniques, and comprehensive randomized studies encompassing cost analyses. These improvements could position the robotic platform as a preferred minimally invasive option for anterolateral segment resections, even in complex cases, along with the integration of technologies like robotics and artificial intelligence in surgical practice [31].

The findings of our study hold significant implications for clinical practice. Our evidence firmly advocates incorporating laparoscopic liver resection as a regular surgical method. Its significant benefits, including decreased bleeding during operations, shorter hospital stays, and lower complication rates, emphasize its capacity to improve patient results and satisfaction. Medical professionals now have the option to explore laparoscopic techniques, especially in cases where it’s viable, like in smaller tumors and less complicated situations. Embracing these methods can lighten the patient load, enabling faster healing, reduced post-surgery discomfort, and improved quality of life.

Additionally, our study underscores the importance of ongoing training and skill development among surgical teams. Ensuring proficiency in laparoscopic techniques can optimize patient results and minimize complications. Furthermore, continuous research efforts are essential to delve deeper into the sources of heterogeneity observed, providing valuable insights for refining protocols and addressing specific challenges in liver surgeries. By incorporating these findings into clinical decision-making processes, healthcare professionals can contribute to a paradigm shift in liver surgery practices, ultimately benefiting patients by offering safer, more efficient, and less invasive procedures.

Our study possesses several strengths that bolster the credibility of our findings. First, we rigorously adhered to rigorous standards outlined in the Cochrane Handbook for Systematic Reviews and PRISMA guidelines, ensuring the robustness of our methodology. Including diverse studies from various countries broadens the applicability of our results, providing a more global perspective. Furthermore, including a substantial number of high-quality RCTs enhanced the robustness of our findings.

However, it's crucial to acknowledge the limitations inherent in our analysis. Variability in the patients in the included studies might have introduced biases, impacting the overall interpretation of the data. Although addressed through appropriate statistical methods, the heterogeneity observed in certain outcomes remains challenging, underscoring liver surgery's complexity. Additionally, excluding studies in languages other than English may have introduced a language bias. Despite these limitations, our study presents a comprehensive overview, offering valuable insights into the comparative effectiveness of LR and OR.

## Conclusions

In conclusion, our analysis recommends the wider adoption of laparoscopic liver resection in clinical practice, particularly for patients where it is feasible. Surgeons should be trained and proficient in laparoscopic techniques to enhance patient outcomes and minimize complications. Future research should delve into understanding the sources of heterogeneity observed in certain parameters, aiming to refine protocols and further optimize the laparoscopic approach. Continuous evaluation and standardization of laparoscopic procedures will contribute to their continued advancement, ensuring the best possible outcomes for liver surgery patients.
